# Endodontic Re-Treatment of Maxillary Second Molar with Two Separate Palatal Roots: A Case Report

**Published:** 2008-07-10

**Authors:** Jamileh Ghoddusi, Abbas Mesgarani, Salman Gharagozloo

**Affiliations:** 1 *Department of Endodontics, Dental Research Center, Dental School, Mashad University of Medical Sciences, Mashad/ Iranian Center for Endodontic Research, Tehran, Iran*; 2 *Department of Endodontics, Dental School, Mashad University of Medical Sciences, Mashad, Iran*

**Keywords:** Anatomic Variation, Case Reports, Second Molar, Palatal Root

## Abstract

Maxillary second molar with two palatal roots is a rare dental anatomy. The diagnosis and treatment of exceeded root may create challenge for clinicians. The authors discuss the retreatment of a maxillary second molar in which exceeded root was undiagnosed in previous treatment. The case report underlines the importance of complete knowledge about root canal morphology which achieved by careful clinical and radiographic examination. In retreatment procedures clinicians should consider missed canals.

## INTRODUCTION

Cleaning and shaping of a root canal system is an essential factor for healing of periapical tissues. Some factors make challenges in root canal treatment. Diagnosis and treatment of teeth with some morphologic variations in roots should be noticed for an acceptable root canal treatment. There are some reports that underline variations in number of roots. Libfeld and Rotstein in a review and radiographic survey of 1200 teeth reported a 0.4% incidence of four- rooted maxillary second molars (1). Beatt reported a case of 5 canals, with 3 in the MB area (2). Christi reported 14 cases of maxillary second molars with two palatal roots in a period of 40 years. He classified them based on root divergency in 3 classes (3). Jafarzadeh and associates reported a maxillary second molar with three-separate buccal roots (4). The following case report describes a maxillary second molar with 2 separate palatal roots and shows the necessity of adequate knowledge about teeth anatomy.

## CASE REPORT

**Figure 1:**
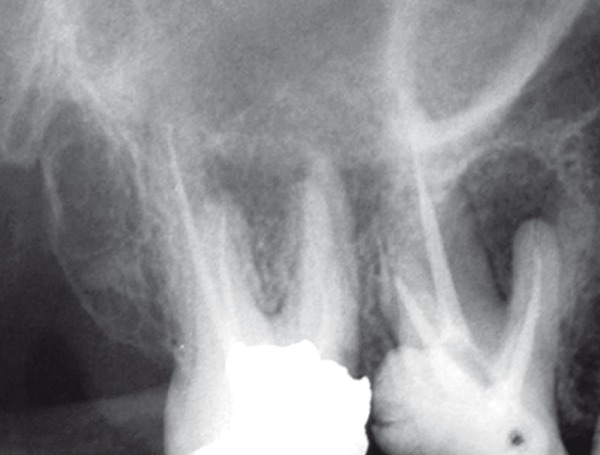
Initial radiograph

**Figure 2:**
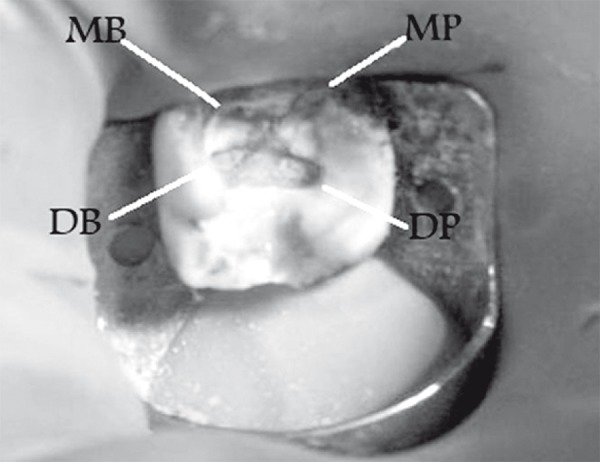
Digital image showing 4 orifices: MB, mesio-buccal; DB, disto-buccal; IVIP, mesio-palat.<tl; DP, disto-palatal

A 45 years old female with a non-contributory medical history was referred to the emergency service of the Dental School of Mashad, Iran because of pain and percussion tenderness on the maxillary right second molar. Clinical examination showed the amalgam filling in the second molar and temporary filling in the first molar. Second molar revealed severe tenderness to percussion. Periodontal pockets were within normal limits. There was no response to heat and cold test on both first and second molar. Tooth mobility was within normal range without any observable swelling. The preoperative radiograph (Figure 1) showed incomplete root canal filling in second molar with 2 untreated roots. The maxillary right second molar was prepared for orthograde endodontic retreatment. The patient received local anesthesia of 2% Lidocain with 1:100000 epinephrine (Daroupakhsh, Tehran, Iran) and the tooth was isolated with rubber dam. The amalgam restoration was removed and during access preparation, previous outline form was extended for locating orifices of two untreated roots (Figure 2); outline form was rectangular and four orifices of mesiobuccal, disto-buccal, mesio-palatal and disto-palatal were located.

**Figure 3:**
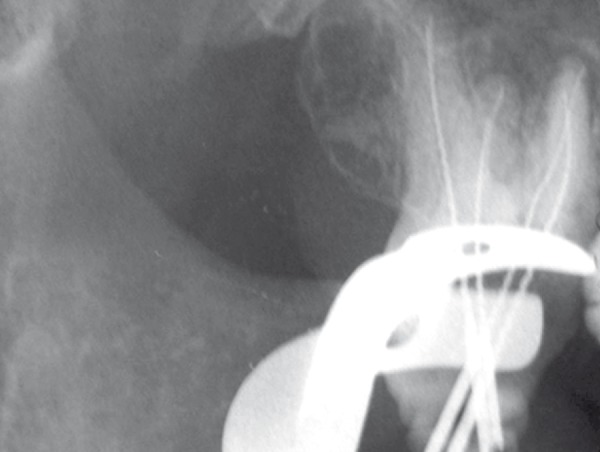
Working length determination

**Figure 4:**
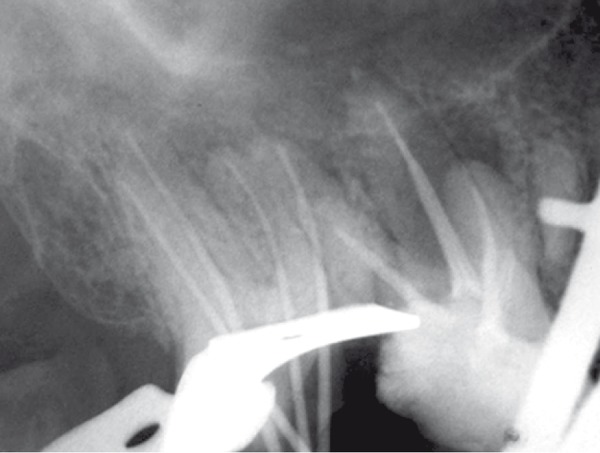
Assessment of master cone

**Figure 5:**
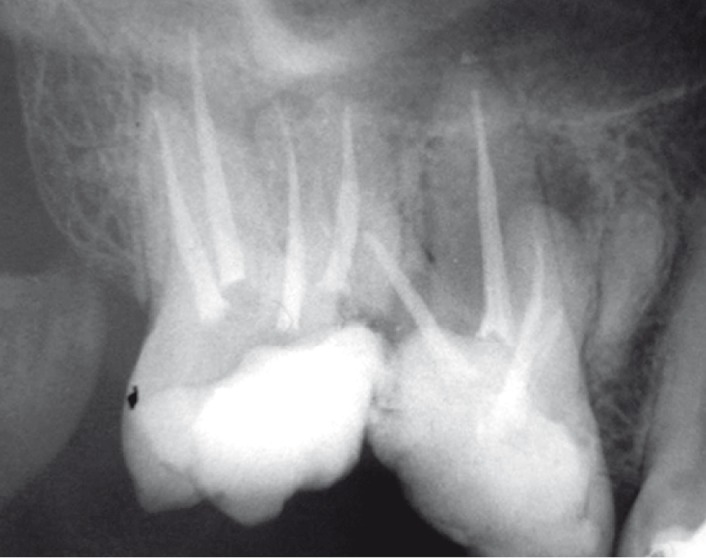
Final radiograph

Gutta-percha was removed from two filled canals with rotary instrument (Mtwo,VDW, Munich, Germany), chlorofonn and Hedstrom files (Maillefer, Switzerland). The working lengths were determined and controlled radiographically (Figure 3). The cleaning and shaping of canals were done by passive step­ back technique and coronal flaring was performed using Gates Glidden Drills No. *2, *3, and 4 (Dentsply Maillefer, Ballaigues, Switzerland). Canals were irrigated with 2.5% NaOCl during instrumentation. After completion of canal preparation, calcium hydroxide paste (Produits Dentaires S.A, Vevey, Switzerland) was placed in all canals with a lentulo spiral (Maillefer, Switzerland) for one week and the pulp chamber was sealed with Cavit (ESPE, Seefeld, Germany). One week later, the canals were obturated with cold lateral condensation technique with gutta-percha cones and AH26 sealer (Dentsply, Konstanz, Germany). Cavit was applied as temporary restoration of the access cavity (Figure4),(Figure 5) and then referred for permanent restoration.

## DISCUSSION

The incidence of 4-rooted maxillary second molar is rare in the literature (3,5). In 2003, Alani reported a case of bilateral four-rooted maxillary second molars that had two buccal and two palatal roots (6). In 2007, Su-Jung-Shin *et al. *reported two cases of second molar with two palatal roots. They suggested using surgical operative microscope to find extra orifices (7). In the present case the distance between two palatal orifices was wider than that of the buccal canals (Figure 2). This finding appears to be similar to cases reported by Alani (6) and Su-Jung-Shin (7). Morphological variations in teeth anatomy must be always considered before beginning the treatment. The case presented showed that missed canals could cause failure of root canal treatment.
